# Pulmonary function testing and chest tomography in patients with acromegaly

**DOI:** 10.1186/2049-6958-8-70

**Published:** 2013-11-13

**Authors:** Gustavo Bittencourt Camilo, Fernando Silva Guimarães, Débora Pedroza Guedes Silva, Roberto Mogami, Leandro Kasuki, Mônica Roberto Gadelha, Pedro Lopes Melo, Agnaldo José Lopes

**Affiliations:** 1 Postgraduate Programme in Medical Sciences, State University of Rio de Janeiro, Boulevard 28 de Setembro, 77, Vila Isabel, Rio de Janeiro 20551-030, Brazil; 2 Rehabilitation Sciences Master’s Program, Augusto Motta University Center, Avenue Paris, 72, Bonsucesso, Rio de Janeiro 21041-020, Brazil; 3 Department of Radiology, State University of Rio de Janeiro, Boulevard 28 de Setembro, 77, Vila Isabel, Rio de Janeiro 20551-030, Brazil; 4 Department of Endocrinology, Federal University of Rio de Janeiro, RuaRodolpho Paulo Rocco, 255, CidadeUniversitária, Ilha do Fundão, Rio de Janeiro 21941-913, Brazil; 5 Biomedical Instrumentation Laboratory, Institute of Biology and Faculty of Engineering, State University of Rio de Janeiro, Rua São Francisco Xavier, 524, PavilhãoHaroldoLisboa da Cunha, Térreo, Sala 104, Rio de Janeiro RJ 20550-013, Brazil

**Keywords:** Acromegaly, Respiratory function tests, Respiratory mechanics, Tomography

## Abstract

**Background:**

Despite the gradual improvement in treatment procedures and cure rates of acromegaly, a steady increase in the mortality rate due to respiratory disease has been documented in recent decades. In this study, our objectives were to describe the abnormalities in lung structure and function that occur in acromegalic patients and to correlate these changes with hormonal levels.

**Methods:**

This cross-sectional study included 20 acromegalic patients and 20 age-and height-matched control subjects, all non-smokers. All subjects underwent spirometry, whole body plethysmography, carbon monoxide diffusing capacity, and respiratory muscle strength. Acromegalic patients also performed high-resolution computed tomography (HRCT).

**Results:**

Most patients were female (65%), with a mean age of 52.5 ± 13 years. Acromegalic patients showed lower values of maximum expiratory pressure (55.9 ± 17.1 vs. 103.7 ± 19.2%; p < 0.001) and maximum inspiratory pressure (71.4 ± 27.8 vs. 85.3 ± 24.1%; p = 0.005) compared to control subjects. The values of forced vital capacity (107.1 ± 15.9 vs. 98.9 ± 21.4%; p = 0.028), total lung capacity – TLC (107.3 ± 12.9 vs. 93.7 ± 7.60%; p = 0.002), residual volume (114.1 ± 22.7 vs. 90.0 ± 14.6%; p < 0.001), and airways’ resistance (3.82 vs. 2.31 cmH_2_O/L/s; *p* = 0.039) were greater in acromegalic patients than in control subjects. The difference between the TLC measured by plethysmography and the V_A_ (alveolar volume) measured during the DL_CO_ maneuver was higher in acromegalic patients than in control subjects (0.69 ± 0.46 vs. 0.19 ± 0.61 L; p = 0.021). The main findings in HRCT in acromegalic patients were air trapping, airway calcification and bronchiectasis, which were observed in 60%, 40% and 35% of cases, respectively. There was no significant correlation between the levels of growth hormone and insulin-like growth factor I, the lung function and the air trapping.

**Conclusions:**

Acromegalic patients show changes consistent with the involvement of the small airways and ventilation inhomogeneity, both in terms of lung function and structure. However, air trapping cannot be explained either by hormone levels or changes in lung function.

## Background

Acromegaly is an endocrine disease that results from the systemic consequences of excessive growth hormone (GH) and insulin-like growth factor I (IGF-I). Its prevalence is approximately 60 cases per million inhabitants, while the incidence is estimated at 3 to 4 cases per million people [1,2]. Patients with acromegaly show a relative risk of mortality from respiratory disease 1.85 times higher than in general population [3]. Excess GH may lead to severe systemic manifestations, including orthopedic, cardiovascular, and respiratory problems [4,5].

The mortality rate due to respiratory disease is approximately three times higher in acromegalic patients than in healthy subjects, and respiratory problems also contribute to approximately 25% of all deaths found in this group of patients [3,6-9]. Patients with acromegaly develop various respiratory disorders resulting from anatomic distortion of soft tissues, cartilage, muscles, and chest and craniofacial bones. This range of abnormalities produces two primary respiratory alterations, known as obstructive sleep apnea and impaired respiratory function [7,10,11].

In acromegaly, the mechanical changes in the respiratory system elastance and the changes in the geometry of respiratory muscles may potentially lead to lung diseases. In these patients, the respiratory muscle strength is altered, the inspiratory time is shortened, the respiratory rate may increase, and subclinical hypoxemia may be present [12]. Despite these alterations, few studies have studied pulmonary function in acromegalic patients [13].

Several studies in acromegalic patients have shown bone and cartilage alterations involving the upper airways and chest wall structures. The axial skeleton is affected in up to 60% patients and includes the thickening of soft and cartilaginous tissues, enlargement of the intervertebral spaces, formation of osteophytes, and kyphoscoliosis [14-16]. The changes in the upper airways and chest wall in acromegaly are already well documented using imaging methods. However, to our knowledge, no research has been conducted to detect lung abnormalities using high-resolution computed tomography (HRCT) in acromegalic patients. Moreover, expiratory computed tomography (CT) has been proposed as a good method for early diagnosis of the involvement of the small airways in systemic diseases [17,18].

Although acromegaly is well studied in the context of cardiovascular, metabolic and musculoskeletal disorders, there is still much controversy regarding the changes occurring in the lungs. The use of HRCT and pulmonary function tests may provide key data on possible pulmonary changes that occur in acromegaly. Thus, our objectives were to describe the abnormalities in lung structure and function in acromegalic patients and to correlate these changes with hormonal levels.

## Methods

### Patients

This cross-sectional study was conducted between June 2012 and March 2013. The study involved 26 subjects with acromegaly (age >18 years) who were followed up at the Clementino Fraga Filho University Hospital of the Federal University of Rio de Janeiro. Diagnoses were based on clinical features and were confirmed by high levels of GH that did not fall below 0.4 ng/mL after an oral glucose tolerance test or IGF-I levels above the upper limit of the age-specific normal range [19,20].

Patients were considered to have controlled acromegaly when their IGF-I levels were within the age-adjusted reference range and when their baseline GH level was less than 1.0 ng/mL [20]. Patients with a history of smoking and patients with comorbidities unrelated to acromegaly that could interfere in the pulmonary function testing were not included in the study. Untreated hypothyroidism and hypocortisolism were also considered as exclusion criteria. A control group of 20 healthy volunteers of both genders was recruited from the Augusto Motta University Center (UNISUAM). These individuals did not exhibit any history of smoking or evidence of cardiorespiratory disorder.

All participants signed an informed consent form, and the protocol was approved by the Research Ethics Committee of the State University of Rio de Janeiro under number 234.362*.*

### Measurements

The serum concentrations of GH and IGF-I were routinely measured using commercial two-site chemiluminescenceimmunometric assays (Immulite, Diagnostic Product Corporation, Los Angeles, CA, USA). The IGF-I level was assayed by IRMA after ethanol extraction of binding proteins (Diagnostic Systems Laboratories, Webster, TX, USA). The percentage of values that exceeded the age-adjusted normal value of IGF-I (upper limit normal value - ULNV) was calculated, and zero was assigned for normal dosages.

The pulmonary function testing consisted of spirometry, body plethysmograph, carbon monoxide diffusing capacity (DL_CO_), and respiratory muscle strength. Measurements were conducted using an HD CPL (nSpire Health, Inc., Longmont, CO, USA), following standard procedures and interpretation [21]. The pulmonary function testing results were expressed as a percent of the predicted values for the Brazilian population [22-25]. Percent of the predicted values outside of the lower limit of normal (LLN) or upper limit of normal (ULN) were considered abnormal. An obstructive ventilatory defect was defined by a forced expiratory volume in 1 second (FEV_1_)/forced vital capacity (FVC) ratio < LLN [26].

The CT images were recorded in a helical CT scanner with 64 channels (Brilliance 40, Philips Medical Systems, Cleveland, OH, USA). The readout time was set to 4 s, with an X-ray tube current of 458 mA and voltage of 120 kVp. Each image acquisition consisted of a block with 250 to 400 2-mm-thick cross sections separated by 1 mm. The images were represented by a square matrix of 768 rows and 768 columns and were recorded without gantry tilt. In all subjects, both end-inspiratory and end-expiratory scans were obtained. Iodinated contrast agent was not used in any of the examinations. The HRCT scans were interpreted by the consensus of two chest radiologists (R.M. and G.B.C.).

### Data analysis

The data distribution was tested using the Shapiro-Wilk test. Comparisons were made using a t-test or Mann–Whitney test. Pearson or Spearman correlation tests were used to assess the associations between variables. The results were expressed as the means and standard deviation or frequencies (percentage). The analyses were performed using the software SigmaStat 3.5 (Systat Software, San Jose, CA, USA). The statistical significance was set at p < 0.05.

## Results

Six out of the 26 initially recruited acromegalic patients were excluded for the following reasons: refusal to participate in the study (4) and inability to reach the acceptability criteria in pulmonary function tests (2). Thus, the acromegalic group included 13 women and seven men with a mean age of 52.5 ± 13 years. Four patients (20%) had hypopituitarism, although they were undergoing hormone replacement therapy and had normal hormone levels. Fifteen patients (75%) underwent surgery and five patients (25%) underwent radiation therapy. As to the pulmonary function, an obstructive ventilatory defect was diagnosed in 40% of the cases. The total lung capacity (TLC) and the residual volume (RV) were > ULN in 15% and 30% of the cases, respectively. Conversely, an RV/TLC ratio > ULN was noted in 40% of the patients. The acromegalic patients’ laboratory data and general characteristics are shown in Table 1.

**Table 1:** General characteristics and laboratory data of 20 acromegalic patients

**General characteristics**	
Age (years)	52.5 ± 13
Gender (female), n (%)	13 (65)
Height (cm)	165 ± 0.10
Weight (kg)	85.8 ± 11.5
BMI (kg/m^2^)	31.7 ± 4.02
Time since diagnosis of disease (months)	101.7 ± 42.6
**Laboratory data**	
GH (μg/L)	2.81 ± 2.78
IGF-I (μg/L)	416.5 ± 290.9
IGF-I (% ULNV)	171.8 ± 115.7

Values are means ± SD or numbers (%). BMI, body mass index; GH, growth hormone; IGF-I, insulin-like growth factor I; ULNV, upper limit normal value.

The control group (15 women and five men) had the following anthropometric characteristics: age = 50.9 ± 15.7 years; height = 162 ± 0.09 cm; weight = 71.3 ± 9.94 kg; BMI = 27.2 ± 3.34 kg/m^2^. There were no significant differences between healthy volunteers and patients with acromegaly regarding age (p = 0.73) and height (p = 0.35). The weight and BMI significantly differed between the two groups (p < 0.001 for both).

Tables 2 and 3 show the comparisons of the pulmonary function parameters between the healthy volunteers and the acromegalic patients (absolute values and percent predicted values). There were statistically significant differences between the values of maximal inspiratory pressure (MIP), maximal expiratory pressure (MEP), FVC, TLC, RV, and airways resistance (Raw). The difference between the TLC measured by plethysmography and the alveolar volume (V_A_) measured during the DL_CO_ maneuver (TLC-V_A_ difference) was also statistically different between the two groups.

**Table 2:** Pulmonary function test results in healthy volunteers and acromegalic patients (absolute values)

	**Control group (n = 20)**	**Acromegaly group (n = 20)**	**p**
FVC (L)	3.27 ± 0.85	3.67 ± 1.03	0.186
FEV_1_ (L)	2.60 ± 0.65	2.92 ± 0.79	0.168
FEV_1_/FVC (%)	84.7 ± 3.25	79.8 ± 4.11	0.598
FEF_25-75%_ (L/s)	2.61 ± 0.71	3.08 ± 1.16	0.130
FEF_25-75%_/FVC (%)	79.4 ± 6.06	83.6 ± 7.93	0.605
MIP (cm H_2_O)	-81.3 ± 29.4	-63.6 ± 28.9	0.063
MEP (cm H_2_O)	121 ± 32.9	92.3 ± 30.79	**0.001**
TLC (L)	4.83 ± 0.98	5.61 ± 1.09	**0.023**
RV (L)	1.55 ± 0.32	2.04 ± 0.38	**<0.001**
RV/TLC (%)	32.6 ± 6.89	37.5 ± 8.94	0.063
R_aw_ (cm H_2_O/L/s)	2.31 ± 0.58	3.82 ± 0.49	**0.039**
DL_CO_ (ml/min/mm Hg)	22.6 ± 5.66	23.3 ± 6.46	0.713
V_A_ (L)	4.61 ± 1.33	4.92 ± 1.19	0.443
DL_CO_/V_A_ (ml/min/mm Hg/L)	5.08 ± 1.06	4.72 ± 0.64	0.205
TLC-V_A_ difference (L)	0.19 ± 0.61	0.69 ± 0.46	**0.021**

Values are means ± SD or numbers (%). DL_CO_, diffusing capacity for carbon monoxide, FEF_25-75%_, forced expiratory flow during the middle half of the FVC; FEV_1_, forced expiratory volume in 1 second; FVC, forced vital capacity; MEP, maximal expiratory pressure; MIP, maximal inspiratory pressure; R_aw_, airway resistance; RV, residual volume; TLC, total lung capacity; V_A_, alveolar volume.

**Table 3:** **Pulmonary function test results in healthy volunteers and acromegalic patients** (% **predicted values)**

	**Control group (n = 20)**	**Acromegaly group (n = 20)**	**p**
FVC (% predicted)	98.9 ± 21.4	107.1 ± 15.9	**0.028**
FEV_1_ (% predicted)	96.2 ± 11.9	104.8 ± 15.5	0.057
FEV_1_/FVC (% predicted)	97.2 ± 3.30	94.0 ± 4.86	0.572
FEF_25-75%_ (% predicted)	91.5 ± 18.9	105.9 ± 20.1	0.261
FEF_25-75%_/FVC (% predicted)	95.2 ± 6.77	99.4 ± 7.05	0.630
MIP (% predicted)	85.3 ± 24.1	71.4 ± 27.8	**0.005**
MEP (% predicted)	103.7 ± 19.2	55.9 ± 17.1	**<0.001**
TLC (% predicted)	93.7 ± 7.60	107.3 ± 12.9	**0.002**
RV (% predicted)	90.0 ± 14.6	114.1 ± 22.7	**<0.001**
RV/TLC (% predicted)	94.9 ± 13.4	107.1 ± 27.2	0.068
DL_CO_ (% predicted)	103.1 ± 13.9	103.7 ± 24.4	0.968
V_A_ (% predicted)	90.5 ± 15.5	93.6 ± 12.2	0.147
DL_CO_/V_A_ (% predicted)	116.4 ± 22.3	110.2 ± 18.5	0.346

Values are means ± SD or numbers (%). DL_CO_, diffusing capacity for carbon monoxide, FEF_25-75%_, forced expiratory flow during the middle half of the FVC; FEV_1_, forced expiratory volume in 1 second; FVC, forced vital capacity; MEP, maximal expiratory pressure; MIP, maximal inspiratory pressure; R_aw_, airway resistance; RV, residual volume; TLC, total lung capacity; V_A_, alveolar volume.

In the group of acromegalic patients, 55% had active disease, while 45% had controlled disease. Although the patients with active disease showed higher mean pulmonary function values in all variables except RV/TLC, no significant differences were found in absolute and percent predicted values between the groups (p > 0.05 for all comparisons).

There was no association between the values of GH, IGF-I (μg/L), and IGF-I (% ULNV) hormone levels and the pulmonary function parameters when using either absolute or percent predicted values (p > 0.05 for all).

**Figure 1:**
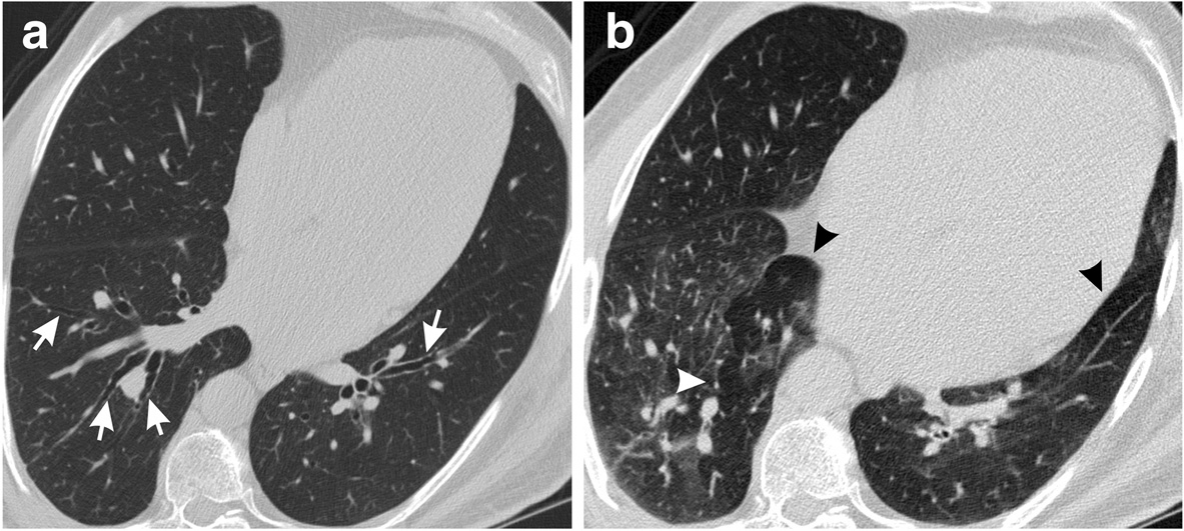
**Bronchiectasis and air trapping in a 56-year-old man with acromegaly who had an RV value of 126% of predicted and a Raw value of 5.23 cm H**_**2**_**O/L/s. (a)** Inspiratory, transverse, thin-section CT scan obtained through the lower lung zone shows bronchiectasis (arrows). **(b)** Expiratory, transverse, thin-section CT scan obtained through the lower lung zone reveals diffuse air trapping (arrowheads).

**Figure 2:**
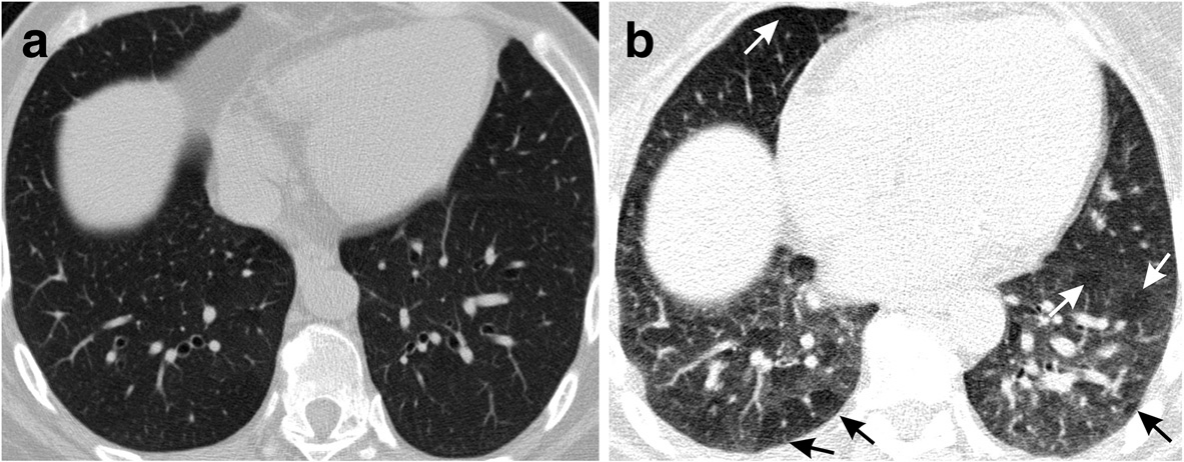
**Air trapping in a 57-year-old man with acromegaly who had a RV value of 173% of predicted and a Raw value of 3.47 cm H**_**2**_**O/L/s. (a)** Inspiratory, transverse, thin-section CT scan obtained through the lower lung zone shows no abnormal findings. **(b)** Expiratory, transverse, thin-section CT scan obtained through the lower lung zone reveals diffuse air trapping (arrows).

**Figure 3:**
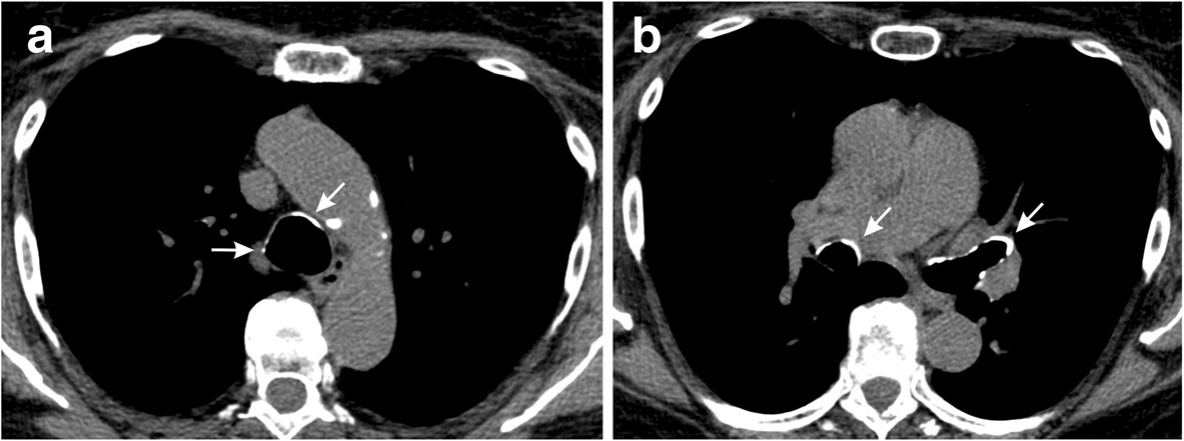
**Airway calcification in a 49-year-old woman with acromegaly who had an RV value of 120% of predicted and a Raw value of 2.95 cm H**_**2**_**O/L/s. (a)** Axial unenhanced CT scan (mediastinal window) shows tracheal calcifications (arrows). **(b)** Axial unenhanced CT scan (mediastinal window) shows bronchial calcifications (arrows).

The main HRCT findings are shown in Table 4. The main change was air trapping (60% of cases), which was diffuse in 20% of the patients (Figures 1 and 2). When sparse, air trapping was mainly detected in the lower lobes. Other common abnormalities included airway calcification (40% of cases) (Figure 3) and bronchiectasis (35% of cases) (Figure 1).

**Table 4:** Abnormalities on chest computed tomography in patients with acromegaly

Air trapping	12 (60)
Located	8 (40)
Diffuse	4 (20)
Bronchiectasis	7 (35)
Ground-glass opacity	4 (20)
Airway calcification	8 (40)
Trachea	6 (30)
Main bronchus	5 (25)

Values are number of cases (%).

No significant difference was found between the mean values of time since disease diagnosis, GH, IGF-I (μg/L), IGF-I (% ULNV), and pulmonary function parameters (absolute values and percent predicted values) when comparing the two groups according to the presence or absence of air trapping in HRCT.

## Discussion

Our study shows that acromegalic patients present with impaired pulmonary function, namely increased static lung volumes, increased TLC-V_A_ difference, and augmented R_aw_. As to the imaging, air trapping is the most common alteration in those patients. In the lungs, the changes in the structure and function mainly affect the lower airways. To date, no study has focused on this issue in a sample of nonsmoking patients without pulmonary comorbidities.

In the present study, pulmonary function results of the two groups were compared both in absolute values and in percent predicted values. Since acromegalic patients tend to present higher values of body weight, body cell mass and extracellular water [27], we consider that the comparison is best seen as percent of predicted values [21]. We observed an increase in FVC (107.1 ± 15.9 vs. 98.9 ± 21.4%; p = 0.028), TLC (107.3 ± 12.9 vs. 93.7 ± 7.60%; p = 0.002), and RV (114.1 ± 22.7 vs. 90.0 ± 14.6%; p < 0.001) in acromegalic patients compared with the control group. The cause of increased static lung volumes in acromegaly has been discussed in the literature. Some studies indicate increased alveolar size [13,28], whereas others consider the increased number of alveoli [29,30]. García-Río et al. [13] noted that the increased lung compliance and reduced lung recoil pressure found in patients with acromegaly were minimized following hormonal control. These data make it unlikely that an increased number of alveoli could explain the increase in lung volume; thus, it would be more plausible to assume an increase in alveolar size in patients with acromegaly.

In the healthy subjects, the V_A_ is essentially the same as the TLC (± 200 mL) and can be used as an estimate of TLC [31]. In the present study, the TLC-V_A_ difference was higher in acromegalic patients than in control subjects (0.69 ± 0.46 vs. 0.19 ± 0.61 L; p = 0.021). Interestingly, the TLC-V_A_ difference provides an estimate of the severity of nonuniform gas distribution, that is the volume of poorly ventilated lung [31,32]. Thus, it may result from large trapped-gas regions in acromegalic patients. It is noteworthy that the TLC-V_A_ difference in patients with air trapping was higher than in those without air trapping on HRCT despite the absence of statistical significance (0.83 ± 0.52 vs. 0.59 ± 0.40 L; p = 0.268).

We noted an increase in R_aw_ (3.82 ± 0.49 vs. 2.31 ± 0.58 cm H_2_O/L/s; p = 0.039) in our sample of acromegalic patients compared with the control subjects, in addition to increased RV. The acromegalic patients also showed higher RV/TLC than the control subjects despite the absence of a statistically significant difference (107.1 ± 27.2 vs. 94.9 ± 13.4%; p = 0.068). Interestingly, abnormalities in acromegalic patients’ lower airways have already been investigated by some authors [10,11]. Harrison et al. [10] noted an increase in R_aw_ in 14 out of 30 acromegalic patients. According to these researchers, the narrowing of small airways can be attributed to vascular congestion of the surrounding lung or even to the accumulation of soft tissues around the small airways. Conversely, Trotman-Dickenson et al. [11] detected lower airway obstruction in eight out of 35 patients. However, both the study by Harrison et al. [10] and that by Trotman-Dickenson et al. [11] included smokers and did not use a control group. Another possible explanation for the involvement of small airways in acromegalic patients is the excessive lung growth because the terminal flows largely depend on lung volume and airways size, and these two variables are interrelated – a phenomenon known as dysanapsis [33,34]. In the present study, however, the mean value of the forced expiratory flow during the middle half of the FVC (FEF_25-75%_)/FVC ratio (a measure to estimate dysanapsis) [33] is greater in acromegalic patients than in control subjects despite the absence of statistical significance (99.4 ± 7.05 vs. 95.2 ± 6.77%; *p* = 0.630). Therefore, it is possible that the highest Rwa in acromegalic patients may only be explained by a loss of elastic tissue of alveoli and small airways.

Acromegaly also affects the function of respiratory muscles [8]. In fact, studies with muscle biopsy have shown segmental muscle fiber degeneration, foci of cellular infiltration, and variable breakdown in both type I and II fibers [35]. We also observed a significant reduction in MEP and MIP values in acromegalic patients compared to normal subjects (55.9 ± 17.1 vs. 103.7 ± 19.2%, p < 0.001; 71.4 ± 27.8 vs. 85.3 ± 24.1, p = 0.005; respectively), corroborating the study by Iandelli et al. [36].

Similarly to other studies [13,37], we also failed to find significant correlation between the level of GH and pulmonary function. In contrast to animal models, no GH binding site has been found in human lungs thus far. This result suggests that this hormone does not play a direct physiological role in alveolar development and growth [38], which could explain our inability to demonstrate any statistically significant correlation.

Alterations in HRCT have not been previously studied in acromegalic patients. Our study showed the high frequency of air trapping (60%) and bronchiectasis (35%). This finding reinforces the notion that the lower airways are somewhat compromised in these patients. Air trapping is induced by narrowing of airways [18] and is associated with bronchiectasis in some cases [39]. Air trapping, when diffuse (which occurred in 20% of our sample) is always associated with some type of pathological alteration, although it may also be found in healthy subjects [18,40,41]. In the present study, we did not observe significant correlations between air trapping and pulmonary function parameters. This suggests that expiratory CT may complement basic evaluations to detect small airways disease in acromegalic patients.

Are there similarities between the 'acromegalic lung’ and the 'senile lung’ from the anatomo-physiological standpoint? Microscopically, lung aging is marked by dilatation of alveoli without signs of destruction of the septa or obvious inflammation [42,43]. In acromegalic patients an increase in alveolar size presumably occurs due to changes in the elastic properties of the lungs, despite the lack of histopathological studies [8,13]. In the elderly, there is increased lung compliance and decreased elastic recoil pressure [44,45]. These findings are also reported in acromegalic patients [13]. The loss of elasticity with aging, along with the loss of support of the small airways, leads to a gradual reduction in the rate of lung emptying [46]. A similar phenomenon presumably also occurs in acromegalic patients, causing changes in the small airways [10,11]. Interestingly, these findings have also been reported in the HRCT of healthy elderly [18,47], similarly to the high frequency of air trapping and bronchiectasis that we have shown in the population of acromegalic patients. Lee et al. [18] found air trapping areas in 76% of subjects aged 61 years or older and also noted a statistically significant correlation between air trapping and age (r = 0.523; p < 0.001). These authors believe that occlusion or luminal narrowing of the airways associated with age may occur at the level of the secondary pulmonary lobule, causing the appearance of air trapping areas. It is noteworthy that airway calcification is a finding that appears in both acromegalic patients and elderly [48]. The changes that occur in lung tissues may at least partly explain the link between the 'acromegalic lung’ and the 'senile lung’. In lung tissue, GH is able to increase the synthesis of collagen type I fibers and mucopolysaccharides [49]. Studies in the elderly show a possible reduction of the amount of elastic fibers in the lung parenchyma and a possible increase in collagen, mainly type III collagen [50].

A critical analysis of the results is warranted. Firstly, we didn’t do more refined measurements for the assessment of small airways, including nitrogen washout. These measurements could give greater support to our hypothesis of early lung aging in acromegalic patients. Secondly, we did not measure the static recoil pressure that would allow a greater understanding of respiratory mechanics in these patients. Thirdly, a pairing with a control group in terms of weight and BMI could help in the interpretation of lung function. Some acromegalic patients were obese, which may have also decreased their lung volumes by reducing respiratory system compliance, precluding finding larger differences with healthy controls. Fourthly, the absence of HRCT in healthy controls, (not permitted by the Ethics Committee), does not allow us to say that the finding of air trapping is characteristic of acromegaly. Notwithstanding these limitations, our results indicate the involvement of the lower airways (not just the upper airways) in acromegalic patients, and this involvement was expressed both in terms of lung function and structure. Thus, this study may serve as a starting point for future anatomical and physiological studies assessing the changes in the lower airways of these patients, including the comparison among active disease, controlled disease and healthy controls in a more robust sample.

## Conclusions

In conclusion, the present study shows that acromegalic patients have abnormalities consistent with the involvement of the small airways and ventilation inhomogeneity, both in terms of lung function and structure. However, the air trapping detected by HRCT cannot be explained either by hormone levels or changes in lung function.

## Competing interests

The authors declare that have no competing interests.

## Authors’ contributions

GBC and AJL conducted literature reviews, and drafted the manuscript. FSG, DPG, RM, and AJL designed the study and helped to draft the manuscript. LK, MRG, PLM, and GBC carried out the study, collected the data and helped to draft the manuscript. All authors read and approved the final manuscript.
